# Economic Analysis of Animal Husbandry Based on System Dynamics

**DOI:** 10.1155/2022/5641384

**Published:** 2022-04-12

**Authors:** Lei Wang, Hongwei Tan

**Affiliations:** ^1^Economics and Management School, Jilin Agricultural University, Changchun 130118, China; ^2^Business School, Changchun Sci-Tech University, Changchun 130600, China; ^3^Language and Culture School, Changchun Sci-Tech University, Changchun 130600, China

## Abstract

In order to improve the effect of animal husbandry economic analysis, this article studies the animal husbandry economy based on system dynamics and studies how to define total factor productivity and its measurement method. Moreover, this article compares and analyzes the production function method, data envelopment analysis, and index method for measuring total factor productivity, selects decision-making units, and determines and processes input-output data. In addition, this article combines the system dynamics model to explore the causal relationship of the animal husbandry economy and builds an intelligent model to intelligently analyze the animal husbandry economy. Finally, this article analyzes the economy and performance of animal husbandry based on simulation experiments. The simulation test results show that the system dynamics model proposed in this article has a good performance in the economic analysis of animal husbandry.

## 1. Introduction

The living standards of residents are constantly improving, the consumption structure is constantly improving, and health awareness is also increasing. Therefore, people's demand for all kinds of healthy and nutritious animal products is growing rapidly, especially the demand for high-quality meat, eggs, and milk products. These shifts in food demand are also adding impetus to the development of animal husbandry. The national economy is a general term for various social production sectors and other labor sectors, including material production sectors such as industry and agriculture and nonmaterial production sectors such as transportation, finance, and commerce. These departments are both interrelated and independent, and together they form an organic whole of the national economy. However, the status of each sector in the national economy is not the same. Among them, agriculture, as the primary industry, plays a fundamental role in the national economy.

In human history, animal husbandry came into being before planting, and the emergence of nomadism separated animal husbandry from agriculture, realizing the first great division of labor in human history, which is a sign of the progress of human civilization. With the development of the times, animal husbandry is showing its important status and significance more and more. Planting and animal husbandry are two extremely important parts of agriculture, and they are interrelated and mutually reinforcing. On the one hand, planting provides essential fodder for the development of animal husbandry. Without the development of animal husbandry, planting is the basis for the development of animal husbandry; on the other hand, the development of animal husbandry provides the fertilizer, power, and funds needed for production, which in turn will promote the development of the planting industry. It can be seen that agriculture is the foundation of the national economy, embodied by both planting and animal husbandry. Both planting and animal husbandry are the basis for human survival and social development.

With the development of society and economy, the status of animal husbandry has become increasingly important, and it is more closely related to the lives of the people. In the food consumption structure of many developed countries, animal products have become the theme of improving people's food structure and nutrition. The proportion of agricultural output value has exceeded that of the traditional planting industry, accounting for about 60%–80%. Driven by consumer demand, the labor force produced by the planting industry has begun to transfer to animal husbandry, which has promoted the industrialization and urbanization of rural areas. The development of animal husbandry has also greatly increased the demand for feed, changed the traditional planting structure, and transformed the traditional dual planting structure based on “grain-economic crops” into “grain-economic crops-feed crops” ternary planting structure has optimized the agricultural industry structure. It is undeniable that animal husbandry has many irreplaceable characteristics of other sectors, such as planting, and its correlation with other industries within agriculture is particularly high. Therefore, the development level of modern animal husbandry has become a measure of whether a country or a region's agricultural industry structure is reasonable. An important sign is the development of agricultural modernization that increasingly requires the support of strong animal husbandry.

This article studies the animal husbandry economy based on system dynamics and constructs an intelligent model to intelligently analyze the animal husbandry economy so as to improve the subsequent development effect of the animal husbandry economy.

## 2. Related Work

Literature [1] believed that due to the development of economic level and the continuous improvement of science and technology, the development of animal husbandry has been greatly impacted, and the traditional development mode of animal husbandry will face the elimination of the market. The application of science and technology to animal husbandry production can improve productivity and reduce costs, improve the ability of animal husbandry to respond to natural disasters or market changes, and promote the sustainable development of animal husbandry. Literature [2] pointed out that animal husbandry is a pillar industry of agriculture, which plays an important role in promoting the level of economic development and improving the comprehensive national strength. Developers should pay attention to the development of animal husbandry, which has an irreplaceable role in the development of the country. Therefore, the production and development of animal husbandry must be rationally arranged, and the construction of animal husbandry infrastructure must be strengthened. Attention must also be paid to fund support and policy guarantees. The sustainable development of animal husbandry is a challenge and an opportunity. Literature [3] found that poultry farms have been affected by the large-scale development of animal husbandry and suggested changing the development model of animal husbandry in order to realize the development of animal husbandry. The scale, standardization, and industrialization of the development model need to meet the needs of the national residents for livestock products and to promote the development of agriculture and rural areas. Literature [4] pointed out that with the adjustment of the production structure of animal husbandry in the early days of the United Kingdom, the production of animal husbandry in the United Kingdom had undergone great changes and analyzed the importance of adjusting the production structure of animal husbandry. Literature [5] pointed out that the education level of the practitioners plays a very important role in the development of animal husbandry.

Literature [6] analyzed the output value and location quotient of modern grassland animal husbandry, studied the industrial advantages and scale, and proposed that the development of animal husbandry should follow the ecological priority, scientific ethics concept, modern pasture system, product differentiation design, synchronization, and the road to related industries. Literature [7] put forward suggestions for improving the development of animal husbandry by studying the coordination of grain production and animal husbandry development, aiming at different problems that arise, such as changing the development model, increasing grain output, and improving feed utilization. Reference [8] used the grey relational method to analyze the relevant data, and concluded that the factor with the highest correlation is the number of Internet users, and finally put forward countermeasures and suggestions to promote the development of animal husbandry. Literature [9] analyzed the output of animal husbandry, the output of animal products, and the number of slaughters and inventories, summarized the problems existing in the development of animal husbandry, and put forward development suggestions such as improving the efficiency of livestock and poultry breeding, improving the breeding system, and strengthening the control of livestock and poultry pollution. Literature [10], based on the analysis of animal husbandry market share, resource endowment coefficient, and product cost, summed up the road of combining agriculture and animal husbandry and industrial clusters, improving the competitiveness of animal husbandry, and promoting the development of animal husbandry. Reference [11] described the specific status of animal husbandry, analyzed the problems that restrict the development of animal husbandry, and put forward suggestions for sustainable development, including sustainable recycling mechanisms, the transformation of development models, and utilization of manure resources. For the development route, in [12], four suggestions for the sustainable development of the animal husbandry economy have been proposed, which are to increase the ideological emphasis on sustainable development of the ecological economy, improve the relevant policy support system for development, and expand the animal product market.

Reference [13] used the production layout index to illustrate the layout of the main production areas of raw milk and used empirical analysis to conclude that the factors affecting the layout change are resources, economy, nonagricultural employment, and the market. Reference [14] put forward suggestions for increasing or decreasing the aquaculture scale in different regions. Reference [15] used the resource endowment index method and the comprehensive comparative advantage index method and used the principle of comparative advantage to research and analyze animal products. Literature [16] compiled and analyzed the relevant data on animal husbandry in the past 30 years, established the livestock and poultry breed structure index, and obtained the evolution process and characteristics of the breed structure index at different stages in the six major production areas of animal husbandry. The dominant varieties in the region are put forward, and countermeasures and suggestions for optimizing the production layout are put forward. Reference [17] used PIL to analyze the evolution process of changes in animal husbandry areas, combined with the principle of comparative advantage, and obtained the direction of layout changes and the advantageous breeding areas. Reference [18] used different indicators to calculate the industrial concentration based on the relevant production data of three years and finally identified the advantageous production areas.

## 3. Economic Calculation Model

DEA method is the most commonly used method in nonparametric frontier efficiency analysis. Farrell first proposed the DEA method, and then Charns further improved the CCR model. With the development of time, many scholars have improved the DEA method to make it more scientific and rational. Now, the DEA method has been relatively mature, and it has become a research method to study input-output efficiency with equal emphasis on traditional econometric methods.

The DEA method is used to scientifically evaluate the decision-making unit and measure the relative efficiency of the measurement unit according to the model. Decision-making unit (DMU) is a specific title; it can be the army, police station, prosecutor's office, or the same entity unit as a supermarket or coffee shop. Moreover, each decision-making unit has the same input and output indicators. DEA calculates input-output data to obtain quantitative indicators of decision-making units and comprehensive efficiency. According to this result, the decision-making units are sorted, the decision-making unit with the largest relative efficiency value is determined, and then the reasons and levels of the ineffectiveness of other decision-making units are analyzed. Finally, it provides suggestions for improvement and development direction of the decision-making unit system. The DEA method can also judge the scale return of each decision-making unit and put forward a scientific suggestion for a decision-making unit to adjust the industrial scale. Compared with other research methods, the DEA method has huge advantages in the research and analysis of multi-input-output systems, which are mainly manifested in the following:

1. It is not necessary to determine the choice of the mathematical form of the production function before the research so as to avoid getting the wrong conclusion because of the wrong function selection;
2. DEA research method does not have to collect price information of input products;
3. In the study of complex systems, the DEA method does not need to sort out the relationship of each subsystem and also does not need to establish the comparability between the indicators;
4. The DEA method does not need to determine the weight of the system and its input and output in advance, excludes the influence of subjective factors, and has the characteristics of simple and objective;
5. DEA sorts out the data from an overall macro perspective to avoid the one-sidedness of the treatment of scattered indicators.

DEA has two models: the CCR model with constant returns to scale and the VRS model with variable returns to scale. CCR is used to calculate comprehensive technical efficiency (STE), including scale efficiency, and VRS is used to calculate technical efficiency (TE) without the effect of scale efficiency.

The CCR model assumes that there are *N* DMUn (1 ≤ *n* ≤ *N*), and each DMU has a kind of input and *b* kind of output, then the DMUn vector is expressed as follows:

(1) $$\begin{array}{c} X_{j}={\left(x_{1j},x_{2j},\ldots ,x_{aj}\right)}^{T}>0,\;j=1,2,\ldots ,N,\\ Y_{j}={\left(y_{1j},y_{2j},\ldots ,y_{bj}\right)}^{T}>0,\;j=1,2,..\ldots N. \end{array}$$

According to the above assumptions, the CCR model formula is as follows:

(2) $$\begin{array}{c} s.t.\left\{\begin{array}{c} \sum_{j=1}^{a}X_{j}\lambda_{j}\leq \theta x,\\ \sum_{j=1}^{a}Y_{j}\lambda_{j}\leq Y_{n},\\ \lambda_{j}\geq 0,\;j=1,2,\ldots ,a. \end{array}\right) \end{array}$$

In this system of equations, the economic meaning of DEA is that DMUn is valid for DEA if and only if *θ*=1 and all inequalities have equal signs. That is, in this system composed of *N* decision-making units, the output obtained by the current input level has reached the optimum. When *θ*=1 and the inequalities of formula (2) are all inequality signs, DMUn is weakly effective; that is, reducing the current input in this system can keep the output level unchanged or increase the output level under the current input level. When *θ* < 1, DEA is invalid; that is, in this system composed of *N* decision-making units, the current output level can be maintained when the current input is reduced by *θ* times.

The CCR model calculates the overall efficiency under the premise that the scale benefit remains unchanged. This assumes that the decision-making unit can increase the output proportionally by increasing the input ratio. This situation is difficult to achieve in actual production, so scholars further study it. By adding a convexity assumption ∑_*n*_*λ*_*j*_=1 to the CCR model, it becomes the aforementioned BBC model for calculating the removal of returns to scale, as shown in the following formula:

(3) $$\begin{array}{c} s.t.\left\{\begin{array}{c} \sum_{j=1}^{a}X_{j}\lambda_{j}\leq \theta x_{n},\\ \sum_{j=1}^{a}Y_{j}\lambda_{j}\geq Y_{n},\\ \lambda_{j}\geq 0,\;j=1,2,\ldots ,a,\\ \lambda_{j}\geq 0,\;j=1,2,\ldots ,a. \end{array}\right) \end{array}$$

By solving the BEC model, we can also obtain the technical efficiency *θ* of each decision-making unit. When *θ*=1, the decision-making unit technology is effective, and formula (3) is used to solve each decision-making unit, and the technical efficiency values of all DMUs are obtained.

Through the CCR and BCC models, we obtained the scale efficiency and technical efficiency of the object under investigation. Next, we will briefly introduce how to calculate pure technical efficiency (PTE). This calculation mainly uses the DEA model with crowded elements:

(4) $$\begin{array}{c} s.t.\left\{\begin{array}{c} \sum_{j=1}^{a}X_{j}\lambda_{j}\leq \theta x_{j0},\\ \sum_{j=1}^{a}Y_{j}\lambda_{j}\geq \frac{1}{\theta }Y_{j0},\\ \sum_{j=1}^{a}\lambda_{j}=1\left(0\leq \theta \leq 1\right). \end{array}\right) \end{array}$$

*θ* obtained in formula (4) is the pure technical efficiency we require. With regard to the introduction of the DEA model, we obtain the technical efficiency, pure technical efficiency, and scale efficiency of the decision-making unit. Lei Ming (1996) stated that management efficiency (ME) can be expressed by the following formula:

(5) $$\begin{array}{c} ME=\text{PTE}\cdot CE\cdot SE\cdot AE. \end{array}$$

If TP is technological progress, then total factor productivity can be derived from the following formula :

(6) $$\begin{array}{c} \frac{\Delta \text{TEP}}{\text{TFP}}=\frac{\Delta TP}{TP}+\frac{\Delta ME}{ME}. \end{array}$$

In traditional economics research, it is usually assumed that the cost is minimized to study the change of total input or that the profit is maximized to study the change of total output. Behind these assumptions, there is a perfectly competitive market, which is very demanding and difficult to achieve in empirical research. The emergence of the distance function perfectly solves the above problems. The distance function is a tool to study multiple input and output without making any assumptions about the production activities of the observer. How Farr et al. utilized the distance function will be described in detail below.

**Figure 1:**
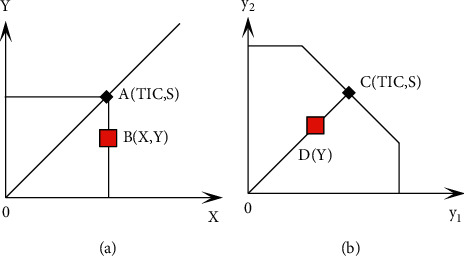
Distance function output. (a) Distance function for a single output. (b) Distance function for double output.

We assume that the vector *X*_*j*_ is the input vector of the DMU and *Y*_*j*_ is the output of the DMU. Because the minimum value of the distance function cannot be determined in the context of multiple outputs, the “inferior bound” is taken as the research object, and the distance function of the output angle is defined as *Do*(*X*, *Y*)=inf{*θ* : (*X*, *Y|θ*) ∈ *P*(*X*)}. P(X) represents all feasible production combinations; that is, input *X*″ can produce *Y*, and *θ* ∈ [0,1] represents production efficiency. When *θ*=1, the resource allocation is reasonable, and when *θ* < 1, it means that the research object is in a state of input redundancy. Under the premise of constant returns to scale, the distance function is single output (Figure 1(a)) or dual-output (Figure 1(b)), as shown in the figure below.

We assume that there is no efficiency loss in production, and the output point should be located at two points AC, but the actual situation may be located at two points BD. Under the premise of constant input, the object at BD two points can increase the output to AC two points; that is to say, from the perspective of output, the distance function represents the limit expansion of output to the production frontier.

**Figure 2:**
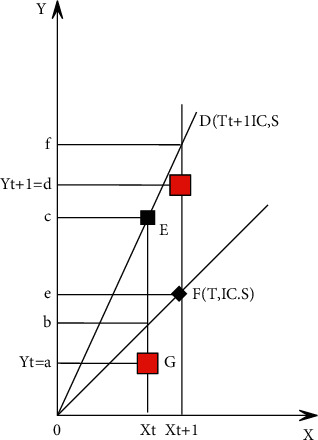
Manquist productivity index from an output perspective.

The functions mentioned above do not take the time factor into account, so we consider the time factor as an independent variable. We assume that the input-output vectors in period *t* and period *t* + 1 are (*X*_*t*_, *Y*_*t*_) and (*X*_*t*+1_, *Y*_*t*+1_), and the distance function diagram of single input and single output is shown in Figure 2. Then the Malmquist exponent with reference to time *t* is as follows:

(7) $$\begin{array}{c} M_{t}=\frac{D_{t}\left(X_{t+1},Y_{t+1}\right)}{D_{t}\left(X_{t},Y_{t}\right)}\\ =\frac{l_{\text{od}}/l_{\text{oe}}}{l_{\text{oa}}/l_{\text{ob}}}. \end{array}$$

The Manquist exponent expressed with reference to period *t* + 1 is as follows:

(8) $$\begin{array}{c} M_{t+1}=\frac{D_{t+1}\left(X_{t+1},Y_{t+1}\right)}{D_{t+1}\left(X_{t},Y_{t}\right)}\\ =\frac{l_{\text{od}}/l_{\text{of}}}{l_{\text{oa}}/l_{\text{oc}}}. \end{array}$$

By borrowing Fisher's method of constructing the ideal index, we take the average of the above two formulas, and we get the Manquist productivity index as follows:

(9) $$\begin{array}{c} M_{t,t+1}={\left(\frac{D_{t}\left(X_{t+1},Y_{t+1}\right)}{D_{t}\left(X_{t},Y_{t}\right)}\times \frac{D_{t+1}\left(X_{t+1},Y_{t+1}\right)}{D_{t+1}\left(X_{t+1},Y_{t+1}\right)}\right)}^{1/2}. \end{array}$$

By deforming formula (9), it can be seen that the total factor productivity change is composed of two parts, the technical efficiency change (EC) and the technical change (TC), that is, the following formula.

(10) $$\begin{array}{c} M_{t,t+1}=\underset{BC}{\underset{︸}{{\left(\frac{D_{t}\left(X_{t+1},Y_{t+1}\right)}{D_{t}\left(X_{t},Y_{t}\right)}\times \frac{D_{t+1}\left(X_{t+1},Y_{t+1}\right)}{D_{t+1}\left(X_{t+1},Y_{t+1}\right)}\right)}^{1/2}}}\cdot \underset{TC}{\underset{︸}{\frac{D_{t+1}\left(X_{t+1},Y_{t+1}\right)}{D_{t}\left(X_{t},Y_{t}\right)}}}. \end{array}$$

When TC > 1, it means technological progress; when TC < 1, it means technological regression; when EC > 1, it means that technical efficiency has improved, and when EC < 1, it means that technical efficiency has declined. The technical efficiency change is composed of pure technical efficiency change (PC) and scale efficiency change (SC); namely, EC = PC × SC, so that TFPch = TC × PC × Sc can be obtained.

## 4. Economic Analysis of Animal Husbandry Based on System Dynamics

**Figure 3:**
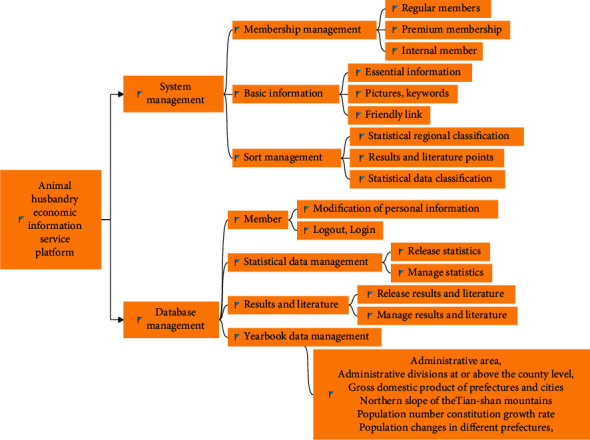
Platform structure diagram.

System management is divided into classification management, membership management, and basic information management. Its main function is to set up the basic information, membership level, and different resource data categories of the entire platform in advance. At the same time, it has the functions of adding, modifying, and deleting, which is convenient for the future function expansion of the platform and the docking with other systems. Data management is mainly divided into animal husbandry economic statistical data management, scientific and technological achievements and literature resource management, and member authority review management. The specific structure of the platform is shown in Figure 3.

**Figure 4:**
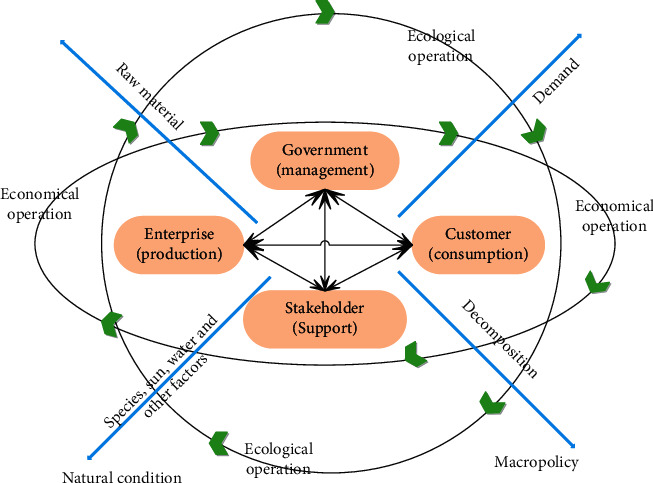
Conceptual structure diagram of animal husbandry ecological economic system.

The ecological economic system of animal husbandry cannot escape the influence of human beings. It is a complex system composed of customers, manufacturers, government, and other stakeholders, and it is reflected in the interrelationship of different subjects. In the era of a self-sufficient natural economy, the production mode of animal husbandry is mainly characterized by the natural regrowth of animals, and it drives the continuation and development of life according to the basic characteristics of the food chain and relies more on natural ecological laws for regulation. The conceptual structure of the animal husbandry ecological economic system is shown in Figure 4.

**Figure 5:**
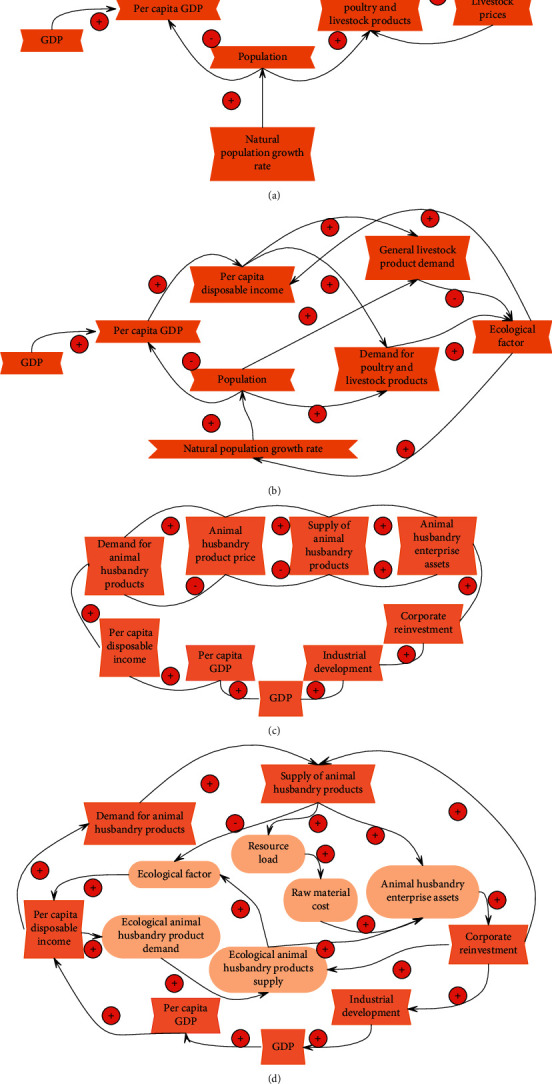
The causal relationship diagram of the animal husbandry economic system. (a) The causal relationship diagram of the demand subsystem from the economic point of view. (b) The causal relationship diagram of the ecological and economic demand subsystem of animal husbandry. (c) The causal relationship diagram of the production subsystem from the economic point of view. (d) The causal relationship diagram of the ecological and economic production subsystem of animal husbandry.

Residents' consumption behavior of livestock and poultry products constitutes the consumption subsystem of the animal husbandry ecological economic system, as shown in Figure 5(a). Residents' demand for livestock products depends on the population and per capita disposable income. The larger the population, the greater the demand for livestock products, and the higher the per capita disposable income, the higher the residents' demand for livestock products. When the supply is in short supply, the price of livestock products will rise, which will in turn affect residents' purchases of livestock products.

Under the current economic conditions, consumers cannot fully accept ecological livestock products but make a balanced choice between general livestock products and ecological livestock products. Therefore, in the consumption subsystem of the animal husbandry ecological economic development system, it includes both the consumption of general livestock products and the consumption of ecological livestock products (as shown in Figure 5(b)), and the two are substitute products for each other.

From an economic point of view (as shown in Figure 5(c)), residents' demand for animal husbandry products prompts animal husbandry enterprises to produce more animal husbandry products to meet the needs of customers. Animal husbandry enterprises can obtain more profits and require more raw materials for production, thus driving the development of raw material enterprises, thereby driving the development of the entire animal husbandry and other related industries, and promoting the GDP of the entire country. At the same time, due to the increase in corporate assets, reinvestment is made to expand production.

With the development of the industry, the pressure from the natural environment continues to increase. The pressure from the natural environment is mainly manifested in the overuse and waste of raw materials and the pollution of the environment by excrement. In order to alleviate the pressure on the environment, it is necessary to invest more balancing funds, which causes the cost to rise, product prices to rise, and consequently the decline of the funds available for the industry to expand and reproduce, thus inhibiting the development of the industry (Figure 5(d)).

**Figure 6:**
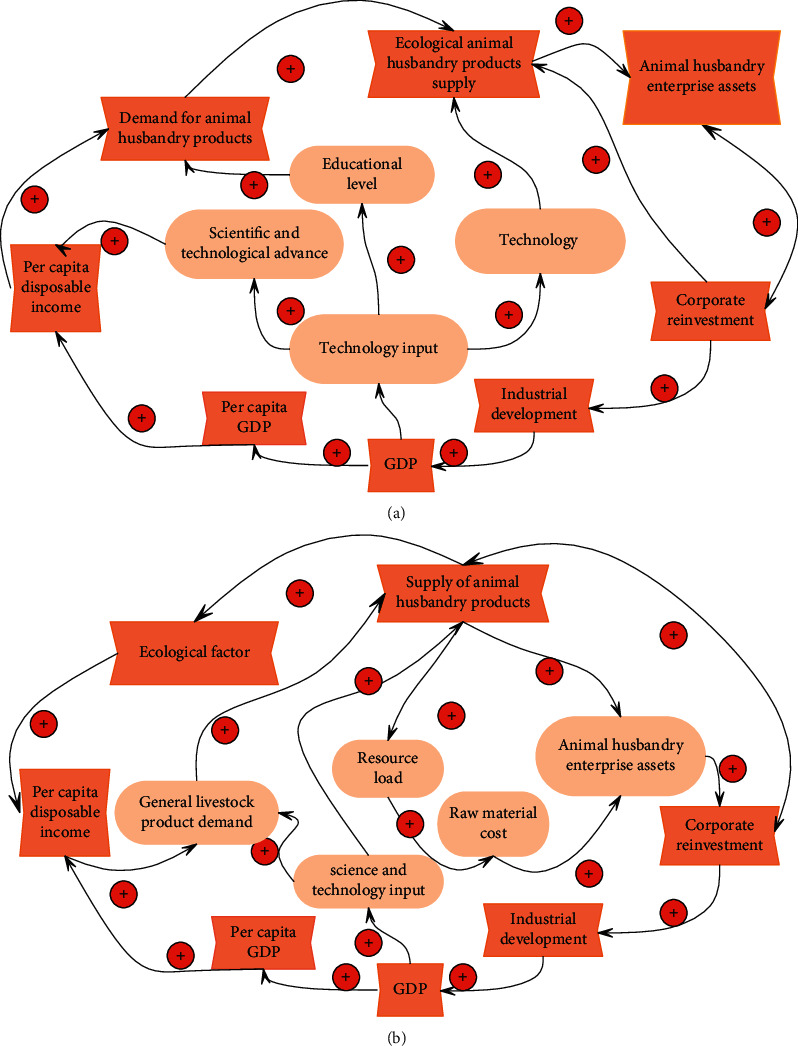
Causal diagram of technical subsystems. (a) The causal relationship diagram of the ecological economic technology subsystem of animal husbandry. (b) Negative feedback relationship diagram of technical subsystem.

As shown in Figure 6(a), the ecological economic technology subsystem of animal husbandry is mainly reflected in three aspects of the consumption subsystem and economic subsystem of animal husbandry ecological economy. First, scientific and technological progress improves the spiritual and material life of human beings, and the increased disposable income of human beings can lead to more consumption of ecological animal husbandry products, which forms positive feedback. The second is the improvement of educational technology and the improvement of residents' educational level brought about by investment in science and technology. Humans realize the significance of ecological animal husbandry products for their own development, which can change consumption awareness and behavior, increase the consumption of ecological animal husbandry products, and form positive feedback. Third, the investment in science and technology has greatly improved the production technology of existing enterprises, improved the production efficiency of enterprises and the utilization rate of resources, and can produce ecological animal husbandry products at a lower cost, forming positive feedback. Conversely, if the technical orientation is wrong, it will exacerbate the negative feedback effect (as shown in Figure 6(b)). For example, the concentration of science and technology on the production and consumption of nonecological products will inevitably increase the capital load and environmental degradation and make the system continue to deteriorate.

**Figure 7:**
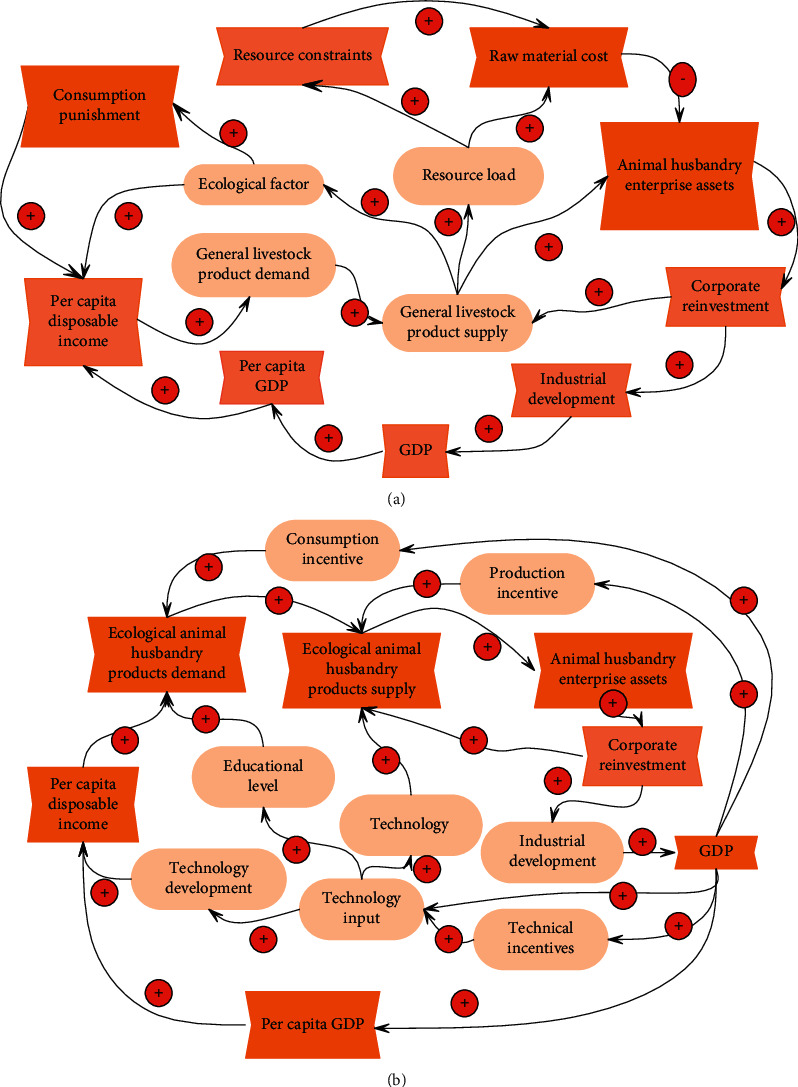
Relationship diagram of the management subsystem. (a) The causal relationship diagram of management subsystems under non-ecological economic behavior. (b) Relationship diagram of management subsystems under ecological economic behavior.

As shown in Figure 7(a), for nonecological economic behaviors, the government sets a higher threshold for customers and producers through economic penalties and direct supervision by regulatory authorities and turns to the production and consumption of ecological livestock products. For example, the government environmental monitoring department sets punitive consumption policies or thresholds according to environmental conditions, which increases the cost of general livestock products and reduces per capita disposable income.

Under the ecological economic behavior (as shown in Figure 7(b)), the government adopts more incentive policies to attract producers, consumers, and technology developers to switch from general animal husbandry products to ecological animal husbandry products, thereby promoting ecological animal husbandry products for nonprofit replacement of ecological livestock products and promoting a virtuous circle of positive feedback.

**Figure 8:**
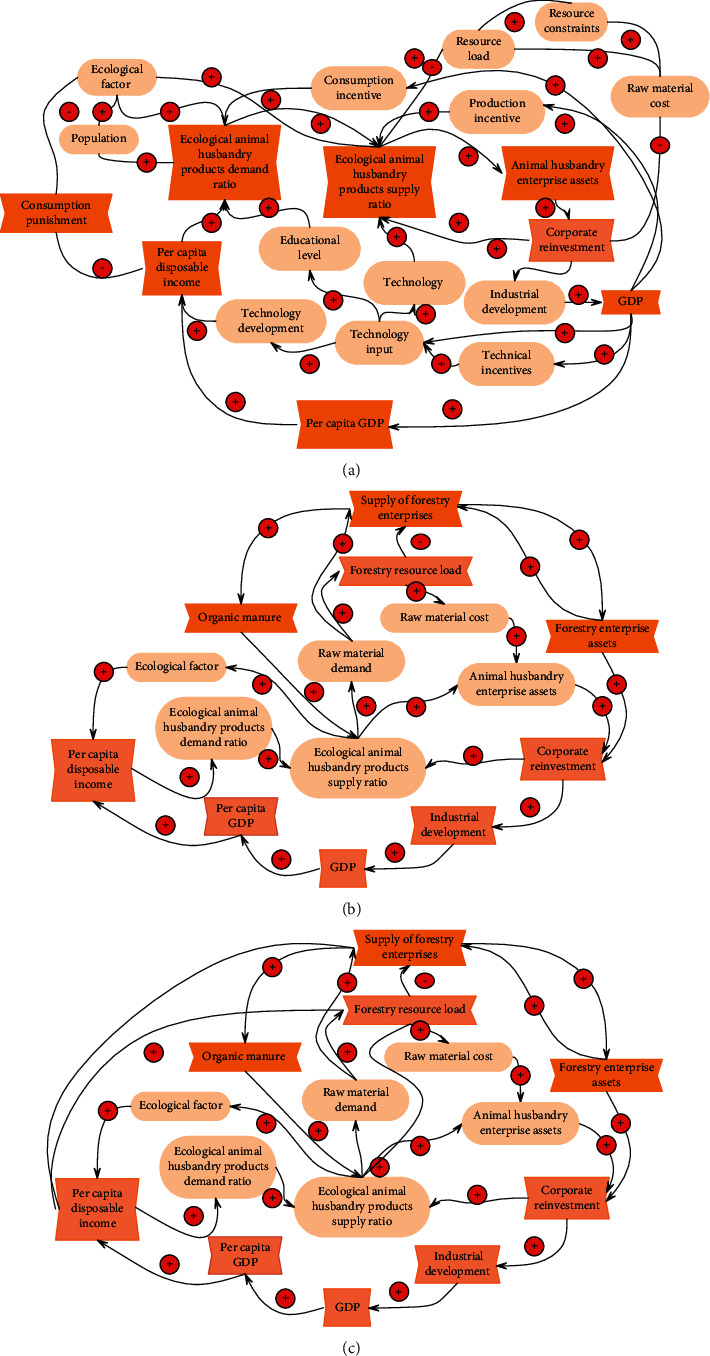
The relationship diagram between the coordinated economic development of animal husbandry. (a) The causal relationship diagram of the ecological and economic development system of animal husbandry. (b) The relationship diagram between the ecological economic development system of animal husbandry and the coordinated development of forestry. (c) The relationship diagram between the ecological and economic development system of animal husbandry and the coordinated development of planting.

In order to further understand the operation law of the animal husbandry ecosystem, we combine nonecological economic products and ecological economic products together. Moreover, this article uses the proportion of ecological animal husbandry products in total animal husbandry products (the ratio of ecological animal husbandry products) to specifically express the choice of consumers, producers, and other subjects for ecological animal husbandry products and ecological behaviors. The SD causal relationship diagram of the whole animal husbandry ecological and economic development system is obtained as shown in Figure 8(a).

Figure 8(b) shows the causal relationship diagram of the coordinated development of the ecological economic system of livestock and forestry. On the one hand, the development of animal husbandry requires forestry to provide more raw materials, so forestry enterprises increase the supply and can obtain more for their own expansion and reproduction. However, due to the constraints of the natural environment, the supply of forestry has a certain load limit, and if the load exceeds the load, it will have a negative impact on human beings and the environment. On the other hand, the development of forestry requires more organic fertilizers, which drives the development of ecological animal husbandry enterprises, while investing in and expanding reproduction. Therefore, the development of forestry also drives the development of the ecological economy of animal husbandry.

Figure 8(c) shows the causal relationship diagram of the coordinated development of the ecological economic system of animal husbandry and crop farming. The relationship between the animal husbandry ecosystem and agriculture basically includes two aspects of coordinated development with forestry. However, unlike forestry, the products of planting not only meet the needs of ecological animal husbandry for raw materials but also meet the needs of people for food. Therefore, human needs aggravate the resource load of the planting industry, so that the ecological and economical operation of animal husbandry is more necessary to balance the needs of human and animal husbandry for the planting industry so as to alleviate and solve the resource load of the planting industry.

On the basis of the above research, the system dynamics model proposed in this article is simulated by the simulation software, and the analysis effect of the system dynamics model on the development of the animal husbandry economy is calculated. The effects in the ecological analysis of animal husbandry economy and the analysis of economic development effects are verified, and the results shown in Tables 1 and 2 are obtained.

It can be seen from the above research that the system dynamics model proposed in this article has a good performance in the economic analysis of animal husbandry and can provide a reference for the development of the animal husbandry economy.

## 5. Conclusion

There are many industries associated with animal husbandry, which influence each other to promote development, and animal husbandry integrates supply, production, and marketing. Among them, the planting industry is responsible for providing raw material feed, livestock and poultry use the feed for growth, and the produced manure is processed into organic fertilizer, which is recycled to the fields for use in the planting industry. The processing industry plays a key role in ensuring the quality of animal products and is an indispensable intermediate link in the output of animal husbandry. The logistics industry ensures the normal transportation and circulation of animal products, and the service industry is indispensable in the process of animal product sales. This article studies the animal husbandry economy based on system dynamics and conducts an intelligent analysis of the animal husbandry economy by constructing an intelligent model. The simulation test study shows that the system dynamics model proposed in this article has a good performance in the economic analysis of animal husbandry and can provide a reference for the development of animal husbandry economy.

## Figures and Tables

**Table 1:** Evaluation of the effect of the system dynamics model in the economic and ecological analysis of animal husbandry.

Num	Ecological economic evaluation	Num	Ecological economic evaluation	Num	Ecological economic evaluation
1	81.51	23	84.57	45	89.92
2	88.18	24	81.88	46	80.16
3	87.69	25	86.58	47	81.01
4	79.07	26	83.75	48	81.21
5	91.45	27	86.56	49	80.95
6	85.30	28	83.66	50	83.49
7	79.55	29	88.37	51	91.37
8	90.74	30	87.66	52	90.22
9	86.98	31	88.34	53	83.76
10	86.44	32	81.35	54	86.13
11	90.42	33	82.38	55	82.03
12	88.07	34	83.03	56	91.79
13	79.89	35	89.97	57	83.04
14	82.71	36	80.46	58	81.62
15	80.73	37	91.15	59	79.22
16	85.29	38	81.65	60	82.57
17	89.71	39	91.59	61	91.07
18	85.28	40	89.22	62	87.26
19	84.68	41	81.25	63	80.71
20	83.35	42	82.16	64	79.54
21	83.67	43	84.09	65	89.17
22	89.09	44	82.62	66	86.21

**Table 2:** Evaluation of the effect of system dynamics model in the analysis of animal husbandry economic development.

Num	Economic development evaluation	Num	Economic development evaluation	Num	Economic development evaluation
1	76.95	23	87.23	45	78.95
2	79.59	24	88.89	46	84.70
3	83.53	25	76.70	47	80.84
4	74.96	26	75.61	48	84.62
5	75.21	27	81.21	49	76.94
6	79.91	28	80.08	50	85.93
7	87.83	29	88.23	51	77.91
8	83.53	30	79.68	52	75.02
9	78.10	31	84.34	53	84.48
10	86.27	32	82.07	54	82.79
11	85.28	33	87.96	55	79.88
12	76.13	34	80.86	56	87.02
13	80.30	35	87.14	57	88.51
14	88.45	36	81.38	58	81.47
15	74.82	37	77.26	59	88.62
16	83.34	38	81.58	60	81.51
17	83.22	39	86.58	61	87.24
18	74.85	40	76.20	62	75.06
19	75.23	41	83.29	63	82.17
20	83.85	42	79.43	64	74.73
21	76.51	43	78.32	65	79.22
22	80.37	44	85.29	66	85.52

## Data Availability

The labeled dataset used to support the findings of this study is available from the corresponding author upon request.
